# Depressive Symptoms and Burnout in Football Players: A Systematic Review

**DOI:** 10.3390/brainsci11101351

**Published:** 2021-10-14

**Authors:** Hugo Sarmento, Roberta Frontini, Adilson Marques, Miguel Peralta, Nestor Ordoñez-Saavedra, João Pedro Duarte, António Figueiredo, Maria João Campos, Filipe Manuel Clemente

**Affiliations:** 1 University of Coimbra, Research Unit for Sport and Physical Activity, Faculty of Sport Sciences and Physical Education, 3040-256 Coimbra, Portugal; joaopedromarquesduarte@gmail.com (J.P.D.); afigueiredo@fcdef.uc.pt (A.F.); mjcampos@fcdef.uc.pt (M.J.C.); 2 Center for Innovative Care and Health Technology, Polytechnic of Leiria, 2411-901 Leiria, Portugal; roberta_frontini@hotmail.com; 3 CIPER, Faculdade de Motricidade Humana, Universidade de Lisboa, 1499-002 Lisbon, Portugal; adncmpt@gmail.com (A.M.); miguel.peralta14@gmail.com (M.P.); 4 Faculty of Health Sciences, Sports Science Program, University of Applied and Environmental Sciences, Bogota 111166, Colombia; sportnestor@gmail.com; 5 Escola Superior Desporto e Lazer, Instituto Politécnico de Viana do Castelo, 4960-320 Viana do Castelo, Portugal; filipe.clemente5@gmail.com

**Keywords:** soccer, sports, depressive symptoms, mental health

## Abstract

The purpose of this article was to systematically review and organise the available literature devoted to the topic of depressive symptoms and burnout in football players. A systematic search was conducted in Web of Science, Scopus, SPORTdiscus, PubMed, and Psychinfo for articles published up to June 2020. The searches yielded 1589 articles, and after the screening process, a total of 18 studies met the eligibility criteria and were included for review. Playing position and conflicts with coach/management seems to have a direct influence on the prevalence of depressive symptoms in current players as do the injuries and life events of former players. During the pre-competition phase, most of the athletes displayed reduced rates, indicating burnout. An exploration of the mental health of football players will help to create models of care and guide professionals so that they may help players achieve better performance while also having better wellbeing. Understanding how to prevent and cope with the emotional wellbeing of football players will be possible to guide players and coaches.

## 1. Introduction

Football is one of the world’s most popular sports. Millions of amateur and professional players are involved. For a player to become an elite performer, they need to have exceptional skills and abilities involving the investment of large amounts of time, effort, and dedication [1].

Football is linked to a multitude of different emotions, some of them experienced very markedly both by fans and players. Hence, there has recently been an interest in understanding the impact of the game on the physical and mental health of fans and players [2].

This is particularly important considering that football puts intense mental pressure on the players, which may increase their susceptibility to certain mental health problems [3]. Losing a game may result in distress, disappointment, sadness [2] and, ultimately, depressive symptoms or even burnout.

Depression is a disturbance of mood characterized by constant feelings of sadness, hopelessness, despair, and a loss of interest in formerly appreciated activities [4]. It may be accompanied by a lack of any desire to move and, thus, with reduced physical activity [5]. A symptom can be defined as the “patient’s perception of an abnormal physical, emotional, or cognitive state” [6] and the depressive symptoms can vary from mild to severe (e.g., feeling sad, changes in appetite, trouble sleeping, loss of energy, feeling worthless or guilty, etc.). According to the Diagnostic and Statistical Manual (DSM) of Mental Disorders (DSM-5), there are several depressive disorders which are characterized by the occurrence of a sad, empty, or irritable mood, and both somatic and cognitive fluctuations that, for the purpose of diagnosis, must significantly affect the individual’s capacity to function [7]. Worldwide, nearly 98.7 million people suffer from depression [4]. Past literature has found that elite sportspeople, such as football players, may present higher levels of depression compared to the general population [8,9]. However, there is little empirical data regarding the mechanisms of depression in football athletes. [10].

The cause of depression and the appearance of depressive symptoms are not fully understood. Apart from biological and genetic predisposition [9], several possible psychological reasons could trigger a previous susceptibility. For footballers, these reasons could include the intense mental demands and the enormous pressure of this particular sport [11], the higher standards of performance, the responsibility of being part of a team, or the fact that, usually, players spend much time away from family and friends [9]. Staying away from home may increase feelings of loneliness and lack of social support which, ultimately, can be related to depressive symptoms [12]. Injuries may also be frequent in elite athletes, and can also play an important role in depression [9]. One must also consider the impact of negative media content [13]. Thus, understanding depression in athletes is crucial not only because of personal suffering, which sometimes leads to desperate acts such as suicide [9], but also because depression is linked to more non-adherence and more dropouts in sport and physical exercise [5].

Burnout is a state of mental, emotional, and physical exhaustion due to a constant commitment to ambitious goals [14]. It has been studied extensively in sports, specifically considering that intensive training has been related to higher levels of burnout [15]. However, burnout has been linked not only to injuries but also to low perceptions of ability in athletes [16]. Thus, higher levels of training, constant injuries and not feeling up to the challenge [15], as well as an inability to get the physical and mental recovery needed may lead to burnout [17].

Whereas burnout and depression share several common features, such as loss of interest, loss of energy or fatigue [7], there has been some discussion whether they are different constructs [18]. Research has found a positive correlation between burnout and depression [19]. This is one of the reasons why most prevention programs with elite athletes focus both on depression and burnout [10]. Although they are usually studied together, recent research recognizes they are distinct concepts [18]. However, the relationship between the two is usually acknowledged and depression in elite athletes seems to be related to several sport-specific mechanisms such as significant stress [20]. In recent years, several studies attempted to better understand the possibly of a biological signature for burnout considering the global research on burnout-depression overlap [21]. Several other studies searched for the psychological mechanisms underlying burnout and depression, suggesting the importance of personality and the importance of the environment where the person is situated [22].

It is also worth mentioning that both athlete burnout and depression are often conceptualized in a stress-based model [10]. In fact, burnout has often been considered as part of depression [23]. It is also important to note that there are no diagnostic criteria to identify and diagnose burnout [24]. Considering the clear association between these two constructs [18], the fact that many football athletes suffer from both burnout and depression and the fact that mechanisms underlying the two variables are not yet understood [10], these two variables should be studied together whenever possible, which may help to clarify the association between the two. Although there have been important systematic reviews regarding the physical health of football players, little research has been devoted to their mental health [25]. Therefore, this review aimed to systematically review and organize the available literature dedicated to the topic of depression and burnout among football players.

## 2. Materials and Methods

This systematic review was conducted according to PRISMA (Preferred Reporting Items for Systematic Reviews and Meta-analyses) guidelines [26]. The study is registered in the International Prospective Register of Systematic Reviews (INPLASY ID 202080074) and DOI number is 10.37766/inplasy2020.8.0074.

### 2.1. Search Strategy: Databases and Eligibility Criteria

A systematic review strategy was conducted according to PRISMA guidelines [26]. Electronic databases (Web of Science, Scopus, SPORTdiscus, PubMed, and Psychinfo) were searched (5 October 2021) for relevant publications before and up to 30 June 2020. A research strategy was performed through Mesh terms obtained at the MeSH Database, leading to the following search strings used (depress* OR “depressive disorder*” OR “depressive symptom*” OR burnout OR “mental health” OR “emotional” OR “emotional depression*”) AND (Soccer OR football). The publications included met the following criteria: (1) to be performed with adult male/female amateur/professional players; (2) contained relevant data concerning depression and/or burnout; and (3) produced relevant data concerning prevalence, treatment, diagnosis, of depression/burnout. Studies were excluded if: (1) they were written in a language other than English; (2) they were editorials, review articles, conference abstracts, books, or book chapters; and (3) they were not subject to peer review. Two reviewers (RF and FC) then independently screened the titles and abstracts of all retrieved studies and determined the eligibility of the potentially relevant full-text articles. If the decision of eligibility was not unanimous, a third reviewer was consulted (HS) to evaluate the identified articles and to reaching a final consensus on inclusion.

### 2.2. Extraction of Data

A data extraction sheet, adapted from the Cochrane Consumers and Communication Review Group’s data extraction template [27], was used to assess inclusion requirements, and was subsequently tested on ten randomly selected studies (i.e., pilot testing). Similar to what was reported above, this process was conducted by two independent reviewers (RF, FC). Any disagreement regarding study eligibility was resolved by a third reviewer (HS). The data extracted from the eligible studies was grouped into three categories: (1) general study descriptors (e.g., authors, year of publication and study design); (2) description of the study population (e.g., sample size, age, gender, country, and competitive level), and (3) data concerning the qualitative synthesis (e.g., outcomes, instruments used to evaluate the symptoms, and main results).

### 2.3. Methodological Quality

An appraisal tool to assess the quality of cross-sectional studies (AXIS) was used to classify the methodological quality of the articles [28]. Additionally, the critical appraisal skills programme checklists were used according to the study design, namely the checklist for: (1) Randomised Controlled Trials; (2) Cohort Studies; and (3) Qualitative studies (http://www.casp-uk.net/ accessed on 15 September 2021).

All the articles related to burnout were cross-sectional. Of the 11 articles related to depression, seven were cross-sectional. The scale includes 20 items, in which one is related to the introduction, 10 are related to methods, five are related to results, two are related to discussion, and two consider other factors. Two of the authors (FMC and HS) independently screened and rated the included full articles. The agreement of both authors was tested using the k agreement rate. The Cohen’s kappa coefficient (k) was executed and revealed a k agreement of k = 0.94.

## 3. Results

### 3.1. Study Identification and Selection

**Figure 1:**
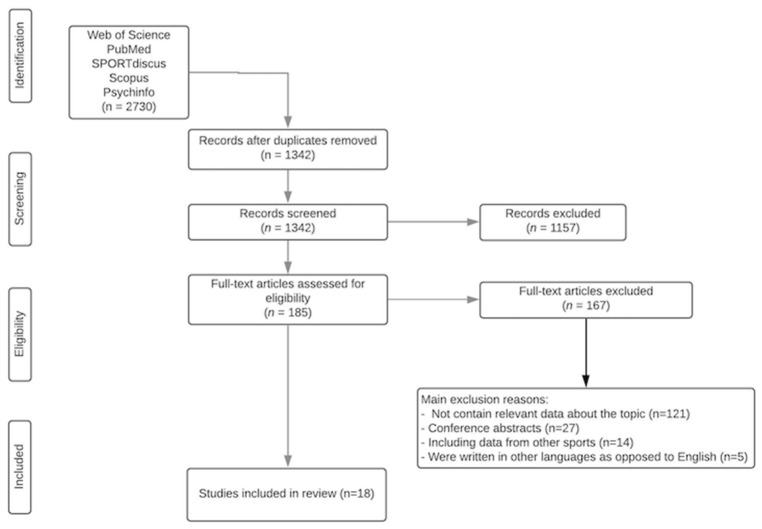
PRISMA flow diagram highlighting the selection process for the studies included in the current systematic review.

The searching of databases identified an initial 2730 titles. These studies were then exported to reference manager software (EndNoteTM X9, Clarivate Analytics, Philadelphia, PA, USA). Duplicates (1388 references) were subsequently removed either automatically or manually. The remaining 1342 articles were screened for their relevance based on titles and abstracts, resulting in the removal of a further 1157 studies. The full texts of the remaining 185 articles were examined diligently. After reading full texts, a further 167 studies were excluded owing to several reasons including a lack of relevance to the research topic (*n* = 121), the fact that they were conference abstracts (*n* = 27), they presented data from other sports (*n* = 14), and they were written in languages other than English (*n* = 5). Following this trimming, 18 articles were accepted for the systematic review (Figure 1).

### 3.2. Quality Assessment

The results of the methodological assessment can be found in Table 1. The overall methodological quality of the studies included in this review, demonstrates that a set of components exists that should be improved in future studies. Approximately 78% of the studies don’t justify the sample size. In 50% of the reviewed papers, measures were not taken to categorise non-responders. Additionally, most of the papers fail in providing information about non-responders.

### 3.3. General Description of the Studies

The characterization of the included studies can be found in Table 2. Among the included studies related to depression and depressive symptoms (*n* = 11), four of them included former players, three included youth players, five included elite/professional players, and two of them included amateur players. Seven of the studies exclusively analysed men, two included both men and women, and two analysed women only. Considering the studies on depression and depressive symptoms, the majority assumed a cross-sectional design (*n* = 7). Considering the studies on burnout (*n* = 7), five of them were focused on elite or professionals and two on youth players. All of the studies (*n* = 7) on burnout were conducted on men and had a cross-sectional study design.

#### 3.3.1. Depression and Depressive Symptoms

The qualitative synthesis of the studies related to depression and depressive symptoms can be found in Table 3. A description of the prevalence of depressive symptoms was the purpose most commonly observed among the studies (*n* = 6). An establishment of the relationship between depression and depressive symptoms and factors that contribute to the occurrence of symptoms was also recurrent among the studies (*n* = 3). One study tested the effect of a controlled intervention to reduce depressive symptoms. Considering the instruments to assess the main outcome, the Center of Epidemiologic Studies Depression Scale was the most used (*n* = 5), followed by the 12-item General Health Questionnaire (*n* = 2).

The prevalence of depressive symptoms found in male and female players as well as in current and former players assumes an important relevance for public health [26,27,28]. Playing position [28,29], and conflicts with coach/management [27] seems to have a direct influence on the prevalence of depressive symptoms in current players, along with injury episodes [30] or live events [28,31] in former players. Mindfulness-based stress reduction programs seem to have a positive effect on retired players [32].

Since terminology is important in the studies developed in this context, Table 4 summarizes the terms adopted by the authors in different studies as well the measures scales used (Table 4).

#### 3.3.2. Burnout

The analysis of burnout in football players has been on the agenda of sport scientists in the last decade (Table 5). All of the studies reviewed are developed in the context of male football, integrating both senior and youth levels as well as the amateur versus professional context. The Athlete Burnout Questionnaire has been used extensively in this context. Only one study used a different scale to analyse burnout symptoms [45]. In general, the reviewed studies always sought to establish relationships between burnout and other moderating variables (e.g., perfectionism, bullying, psychological need satisfaction, organizational stressors, self-determined motivation, and leader-member exchange).

## 4. Discussion

### 4.1. Depression and Depressive Symptoms

The prevalence of depressive symptoms was analysed in current and former players (both men and women). Depressive symptoms varied from 16.7% [27] to 39% [33]. Severe symptoms were found in between 14% [30] and 33% [37] of players with depressive symptoms. Understanding the prevalence of depressive symptoms is important for public health. The prevalence found in this review suggests that depressive symptoms are more prevalent in football players when compared to the general population [9]. However, studying the prevalence of depression may be very difficult since the definition of depression ranges from episodes of unhappiness to persistent mood changes [47]. In the case of current players, reviewed studies suggested the influence of playing position as a possible cause for variation in depressive symptoms [37,38]. Thus, and considering that some research highlighted that some playing positions (such as goalkeepers) used to have higher levels of depressive symptoms, future studies should try to better explore the role of playing position. Table 2 and Table 3 present more detailed information regarding the samples assessed as well as the instruments used to assess depressive symptoms. It is important to state that the variability in prevalence estimates may be due to different assessment methods, times or even samples (e.g., difference prevalence in gender etc.) [10]. Systematic reviews such as the one we present may bring some light and help to better understand what has been made in this field.

Some of the studies have tried to justify the causes of such prevalence. In the case of former players, the main reason to justify depressive symptoms was having to retire because of injury [30] or live events [35,39]. In the case of current players, some reports suggested the influence of conflicts with the coach/management [37], or live events [39]. Interestingly, in one study, no differences were found in depressive symptoms between injured and non-injured players [32]. Nonetheless, injury has been highlighted in past literature as being linked with depression [9]. Thus, we acknowledge the need of future studies to continue exploring the link between injuries and depressive symptoms. Moreover, when a player gets injured, it must be a priority to connect with that player and try to understand if he/she needs some extra support to deal with any subsequent mental issues related to the injury. Regarding former players, it is also important that, at the end of their career, they receive support in terms of a retirement plan.

A randomized clinical trial [29] tested the effects of a mindfulness-based stress reduction programs on retired players. The intervention revealed a significant benefit compared to the control group. Additionally, the effects continued through to the follow-up. Cognitive-behavioural therapies are still the most used in sports psychology interventions and football interventions [48]. Mindfulness strategies, focusing on the present in a non-judgmental way [49], may be helpful. However, future studies should try to understand if other interventions and strategies might be effective in order to find the best methods to prevent and alleviate depressive symptoms in football players. Although many studies have already discussed some of the most effective interventions in terms of fighting depression and depressive symptoms, football players are a specific population, and their uniqueness should be considered.

Finally, a result worth noting relates to sex differences. In one study, no significant differences were found between sexes [23]. However, Junge and Feddermann-Demont [29] reported significant differences between sexes, with females presenting higher prevalence of depression. This result is also present in other populations [50] and it is not exclusive to football players.

### 4.2. Burnout

The reviewed studies demonstrated the existence of a great dispersion of objectives. In this sense, the study of Verardi et al. [41] demonstrated that during the pre-competition phase, most of the athletes displayed reduced rates, indicating burnout. Additionally, it is important to note that professional players achieved maximum average scores related to burnout in the three dimensions, which should be taken into consideration in future studies.

The other studies included in this review always sought to establish relationships between burnout and other moderating variables, namely perfectionism [42], bullying [40], psychological need satisfaction [43], organizational stressors [44], self-determined motivation [46], and leader-member exchange [45]. Football players have very busy lives, with different competitions sometimes in different parts of the world. Players should take some time to switch off and both physically and mentally detach from the game to prevent burnout symptoms [9]. Considering the association that burnout may have with so many different variables, it is important to better understand the mechanisms underlying them. Coaches, with their privileged position, should be alert to earlier signs of burnout, such as physical difficulties, sadness, or even fatigue. In some cases it may be possible to schedule individualized training sessions. Working closely with a psychologist may also be important. Coaches might identify players needing attention to a sports psychologist. The sports psychologist could then work with the player in order to help him/her overcome any difficulties.

Specific techniques may apply, depending on the particularities of burnout. Nonetheless, typical techniques that may help reduce anxiety may also be used with these players, such as: imagery techniques, relaxation, and problem-solving techniques. Considering the association found between anxiety and burnout [51], it is possible that techniques to lower anxiety in football players may be effective in helping players deal with burnout as well. One important specific finding of one of the papers was the mediating role of psychological need satisfaction in the relationship between harmonious passion and dimensions of athlete burnout. This suggests that harmonious passion may protect athletes from the development of athlete burnout through psychological need satisfaction [43]. This finding has many important practical implications that are discussed in the paper, such as the importance of promoting sporting atmospheres emphasizing harmonious tendencies. Creating a supporting environment may also be a protective factor against bullying, which has also been found to be associated with player burnout [40].

It is important to note that from the studies selected for the review that met the inclusion criteria, only one study [31] referred to both depression and burnout. This is also an important result. Considering the importance that researchers have attributed to both variables [23] and the fact that prevention programs in elite athletes usually highlight the fact that it is crucial to consider both variables [10], the results of the present study underline that there is still work to be done in the area. In fact, the literature has already pointed to the importance of studying both variables, especially considering that that mechanisms underlying the two variables are not yet understood [10]. The results of the present study reinforce that studies underlying the connection between the two variables are still scarce.

### 4.3. Recommendations and Future Directions

The studies found in this review revealed some interesting results in terms of practical implications and possible future directions in research. However, there is still a long way to go.

First of all, the results highlight the importance of talking about depression and depressive symptoms. Depression is a serious disease, and the results of this review suggest that the prevalence of depressive symptoms in football players is very high. It is imperative to talk about depression, the symptoms, and the consequences, as well as the early signs of burnout. Improving open communication between players and football staff may be important. It is essential not to hide it, especially considering that depression and depressive symptoms can often be linked to irreversible issues such as suicide [52]. However, there is still a lot of stigma surrounding depression. Many people are afraid to seek help, concluding that they will be judged as weak. Mental health is usually relegated to another dimension when compared to physical health. Thus, an athlete seeking help for a physical condition is usually not stigmatized the same way they feel they might be for seeking help for mental health problems. Educational programs at the beginning of a career, and possibly at the end of a career, might help players to understand the early signs of the disease, the places where they may seek help, and the best times to do so.

Moreover, clinical sport psychological approaches are still scarce [11]. Considering the prevalence of depression and burnout in football players, it is of the utmost importance to incorporate psychologists in multidisciplinary teams working with these professionals. Only a sports psychologist has the necessary tools to help overcome depressive symptoms and burnout symptoms. Thus, multidisciplinary teams, with a psychologist, would provide a balance between physical and emotional components, using important tools and techniques that would help to prevent burnout and depression in players [17]. It is important to note that psychologists may work with the team, but also in particular cases with particular players. A counselling-based session may, for instance, be needed in particular cases and for specific players. In fact, due to the prevalence revealed in the studies, psychotherapeutic support during one’s football career may be needed. Although coaches are not experts at diagnosing symptoms, considering their privileged position and proximity to players, they should be alert for possible symptoms and early signs. A routine screening of mental health problems (and specifically a quick screening of depression, depressive symptoms and burnout), performed by a psychologist, may also be important. Future studies should also try to understand if players are usually referred to psychologists/psychiatrists to decrease possible suffering and, if so, the strategies that were used (and to what level of effectiveness).

The study’s result demonstrated the existence of a great dispersion of objectives on studies. Thus, more studies are needed. It is imperative to develop a comprehensive understanding of the mental health of football players and to create models of care and management which will have an impact on the performance of players and their wellbeing [25]. By better understanding how to manage the emotional wellbeing of football players, it will be possible to guide all sports staff, players and coaches alike [53]. Coping skills should also be researched extensively in terms of working toward a better understanding of optimal educational intervention.

Another important recommendation relates to social support. Considering the nature of football, and the constant travel and possible disillusions related to the game, social support may be an important preventive strategy. Social support may also be an important protective factor [13], helping players who are dealing with critical problems such as injury and loneliness [9,12], and minimizing important negative behaviours such as bullying.

Finally, future investigations should take into account the level of professionalism of footballers. The evolutionary tendencies of the game (e.g., compressed schedules and multiple games in short amounts of time) can have a significant influence on depressive symptoms and burnout.

Future studies should consider not only the use of self-reports but also more accurate methodologies. Self-report measures are of utmost importance in screening and measuring progress. However, when we are interested in studying the diagnosis of diseases such as depression it is important to include a professional structured clinical interview by professional psychiatrists/psychologists and to have a confirmed diagnosis.

A possible limitation of this systematic review is that it only includes studies in English from the Web of Science, Scopus, SPORTdiscus, PubMed, and Psychinfo databases. Additionally, the fact that the selected studies are mostly cross-sectional may result in a limitation of the existing research to date, along with the level of evidence (as explained in Section 3.2). Additionally, the lack of homogeneous studies about the topic under study should be considered a limitation that future research should take into account. It is also important to note that the use of self-reported scales do not allow the generalization of some of the results.

## 5. Conclusions

Although there are many studies in this area, little attention has been paid to the psychological component. When looking for information about depression, and depressive symptoms or burnout and sport, there is a clear primacy with respect to the beneficial effects of sports and exercise on symptoms of depression and anxiety, while there is little on the levels of depression and burnout of players. Recently, however, there has been a greater openness to this topic.

The reviewed studies showed that depressive symptoms in football players are more prevalent when compared to the general population. During the pre-competition phase, most of the athletes displayed reduced rates of indicating burnout. Considering the constant travel and possible disillusions related to the game, social support might be an important preventive strategy and protective factor. This type of intervention would help players dealing with critical problems such as injury and loneliness, while serving to minimize a host of negative behaviours. By better understanding how to prevent and cope with the emotional wellbeing of football players, it will be possible to guide all sports staff.

## Figures and Tables

**Table 1:** Quality assessment of individual studies.

Study	1	2	3	4	5	6	7	8	9	10	11	12	13	14	15	16	17	18	19	20
Depression/Depressive symptoms																				
Norouzi et al. [29]	Y	Y	Y	N	N	N	Y	Y	Y	Y	Y	Y	Y	--	--	--	--	--	--	--
Junge and Prinz [30]	Y	Y	N	Y	Y	Y	N	Y	Y	Y	Y	Y	N	N	Y	Y	Y	N	N	Y
Smith et al. [31]	Y	Y	Y	Y	N	N	Y	Y	Y	Y	Y	Y	Y	Y	--	--	--	--	--	--
Olmedilla et al. [32]	Y	Y	N	Y	Y	Y	N	Y	Y	Y	Y	Y	N	N	Y	Y	Y	Y	N	N
Jensen et al. [33]	Y	Y	N	Y	Y	Y	N	Y	Y	Y	Y	Y	N	N	Y	Y	Y	Y	N	N
Wood et al. [34]	Y	Y	Y	Y	Y	Y	Y	Y	Y	Y	--	--	--	--	--	--	--	--	--	--
Van Ramele et al. [35]	Y	Y	Y	Y	N	N	Y	Y	Y	Y	Y	Y	Y	Y	--	--	--	--	--	--
Sanders and Stevinson [36]	Y	Y	N	Y	Y	Y	N	Y	Y	Y	Y	Y	N	N	Y	Y	Y	Y	N	Y
Prinz et al. [37]	Y	Y	N	Y	Y	Y	N	Y	Y	Y	Y	Y	N	N	Y	Y	Y	Y	N	Y
Junge and Feddermann-Demont [38]	Y	Y	Y	Y	Y	Y	Y	Y	Y	Y	Y	Y	N	Y	Y	Y	Y	N	N	Y
Gouttebarge et al. [39]	Y	Y	Y	Y	Y	Y	Y	Y	Y	Y	Y	Y	N	N	Y	Y	Y	Y	N	Y
Burnout																				
Yildiz [40]	Y	Y	N	Y	Y	Y	Y	Y	Y	Y	Y	Y	N	N	Y	Y	Y	Y	N	N
Verardi et al. [41]	Y	Y	N	Y	Y	Y	N	N	Y	Y	Y	Y	N	N	Y	Y	Y	N	N	Y
Hill [42]	Y	Y	N	Y	Y	Y	Y	N	Y	Y	Y	Y	Y	Y	Y	Y	Y	Y	N	Y
Curran et al. [43]	Y	Y	N	Y	Y	Y	Y	Y	Y	Y	Y	Y	N	N	Y	Y	Y	Y	N	Y
Tabei et al. [44]	Y	Y	Y	Y	Y	Y	Y	Y	Y	Y	Y	Y	N	N	Y	Y	Y	Y	N	Y
Yildiz [45]	Y	Y	N	Y	Y	Y	Y	Y	Y	Y	Y	Y	Y	N	Y	Y	Y	N	N	N
Curran et al. [46]	Y	Y	N	Y	Y	Y	Y	Y	Y	Y	Y	Y	Y	N	Y	Y	Y	Y	N	Y

Abbreviation: Y, yes; N, no.

**Table 2:** Characterization of the included studies.

Study	TS	N	CL	Sex	Age	Years of Experience	Country
Depression/Depressive symptoms							
Norouzi et al., 2020 [29]	RCT	40	Retired	Men	34.1 ± 1.7	N.R.	Iran
Junge and Prinz, 2019 [30]	CS	290	Elite and amateur	Women	<20 to >26	N.R.	Germany
Smith et al., 2018 [31]	Cohort	108	Youth	Men	16.2 ± 1.8	3.65	United Kingdom
Olmedilla et al., 2018 [32]	CS	187	Amateur levels	Men and women	22.1 ± 4.7	N.R.	Spain
Jensen et al., 2018 [33]	CS	323	Junior and professional	Men	22.1 ± 5.2	N.R.	Denmark and Sweden
Wood et al., 2017 [34]	DQ	7	Professional	Men	NR	N.R.	England
Van Ramele et al., 2017 [35]	Cohort	194	Retired	Men	35	12 *	International
Sanders and Stevinson, 2017 [36]	CS	307	Retired	Men	46.8 ± 15.7	6.7 *	United Kingdom
Prinz et al., 2016 [37]	CS	157	Elite	Women	33.0 ± 6.25	N.R.	Germany
Junge and Feddermann-Demont, 2016 [38]	CS	471	Elite and youth	Men and women	League men: 24.8 ± 2.3; U-21 men: 18.4 ± 1.2; League women: 21.0 ± 3.8	N.R.	Switzerland
Gouttebarge et al., 2015 [39]	CS	253	Current and retired	Men	Current: 27 ± 5; Retired: 36 ± 5	9 and 12 *	International
Burnout							
Yildiz, 2015 [40]	CS	102	Professional	Men	25.55	6.72 *	Turkey
Verardi et al., 2015 [41]	CS	134	Professional and amateur	Men	22.8 ± 4.0 and 17.1 ± 0.8	NR	Brazil
Hill, 2013 [42]	CS	171	Elite	Men	16.17 ± 1.57	4.35	England
Curran, et al., 2013 [43]	CS	173	Elite	Men	15.46 ±1.47	9.45	England
Tabei, et al., 2012 [44]	CC	98	Youth	Men	20.25 ± 1.20	13.22	England and Japan
Yildiz, 2011 [45]	CS	150	Elite	Men	25.7 ± 4.40	7.07	Turkey
Curran, et al., 2011 [46]	CS	149	Youth	Men	16.2 ± 2.00	9.1	England

Abbreviation: TS, Type of study; N, number of participants; CL, Competitive level; RCT, randomized clinical trial; CS, cross-sectional; DQ, descriptive qualitative; NR, non-reported; *: professional years. U-: under.

**Table 3:** A qualitative synthesis of the studies related to depression and depressive symptoms.

Study	Aim	Outcomes	Instrument	Main Results
Norouzi et al., 2020 [29]	Test the efficacy of a mindfulness-based stress reduction program on depression symptoms	Depressive symptoms	Montgomery-Åsberg Depression Rating Scale	Depressive symptoms significantly decreased after an intervention (22.9 to 9.4 points) and the values in follow-up also remain low (12.4 points). The intervention group had significant benefits compared to the active control group.
Junge and Prinz, 2019 [30]	Describe the prevalence and risk factors of depression	Depressive symptoms	Center of Epidemiologic Studies Depression Scale	The prevalence of depressive symptoms was 31% among the participants. Additionally, 14% of the players revealed severe symptoms of depression. Despite 16% declared the need for clinical support, only 1/3 have reported that are under treatment or counselling.
Smith et al., 2018 [31]	Test relationships between depressive symptoms, burnout, and perfectionism	Depressive symptoms	Center of Epidemiologic Studies Depression Scale	Socially prescribed perfectionism did not predict depressive symptoms. However, depressive symptoms did predict an increase in socially prescribed perfectionism over time.
Olmedilla et al., 2018 [32]	Analyse the post-injury impact on depression	Depressive symptoms	Depression, Anxiety and Stress Scale—21 Items	No significant differences were found between sexes. Depression symptoms were not significantly different between injured and non-injured players.
Jensen et al., 2018 [33]	Analyse the relationship between perfectionism and depressive symptoms	Depressive symptoms	Center of Epidemiologic Studies Depression Scale	A prevalence of 16.7% was found among the participants. Depression was not correlated with age. However, significantly greater values of depression were found in youth than in professionals.
Wood et al., 2017 [34]	Describe the lived experiences of mental health difficulties	*	*	Survival terms emerged from the interviews. Injury and transition were related to mental health difficulties.
Van Ramele et al., 2017 [35]	Analyse the incidence of anxiety/depressive symptoms	Depressive symptoms	12-item General Health Questionnaire	Common mental disorders ranged between 11 and 29% during 12-month. Players with life events showed a higher risk of experiencing mental disorders.
Sanders and Stevinson, 2017 [36]	Analyse the relationships between career-ending injury, chronic pain, athletic identity and depressive symptoms	Depressive symptoms	Short Depression-Happiness Scale	Retired players with depressive symptoms were more likely to cite injury as retirement reasons. The injury was the greater determinant to explain the depressive symptoms in retired players.
Prinz et al., 2016 [37]	Analyse depressive symptoms during and after career	Depressive symptoms	Modified Centre of Epidemiologic Studies Depression Scale	Almost 1/3 of the participants had symptoms of major depression at least once during their career. Average depression scores were different between playing positions and levels of play. Conflicts with coach/management were frequently stated as a reason for lows in mood.
Junge and Feddermann-Demont, 2016 [38]	Analyse the prevalence of depression	Depressive symptoms	Centre of Epidemiologic Studies Depression Scale	Players had a similar prevalence of depressive symptoms to the general population, despite under-21 reported higher prevalence. Symptoms of severe depression were identified in an average of a player per team. Age, sex, playing position, level of play, and a current injury resulted in significant differences in depressive symptoms.
Gouttebarge et al., 2015 [39]	Analyse the prevalence of anxiety/depression	Anxiety/depressive symptoms	12-item General Health Questionnaire	The prevalence of mental health problems achieved 26 and 39% for current and former players, respectively. The low social support and recent live events were cited as main reasons to justify the mental health problems.

* qualitative methodology (interview).

**Table 4:** Definitions used in the depression variable and evaluation instrument tools.

Study	Depression Variable	Measurement
Norouzi et al. [29]	- The authors define the variable as “depressive symptoms”. However, during the paper, they also use the term depression. - The sum scores can be interpreted as follows (Ahmadpanah et al., 2016): 0–6 points: no depression; 7–19 points: mild depression; 20–34 points: moderate depression; >34 points: severe depression.	- Montgomery-Åsberg Depression Rating Scale (MADRS)
Junge and Prinz [30]	- The authors define the variable as “depressive symptoms”. However, during the paper, they also use the term depression. - The questionnaire also included questions on frequency of intake of medication for depression.	- The Center for Epidemiologic Studies Depression Scale (CES-D)
Smith et al. [31]	- The authors define the variable as “depressive symptoms”. However, during the paper, they also use the term depression.	- The Center for Epidemiologic Studies Depression Scale (CES-D)
Olmedilla et al. [32]	- Depression	- DASS-21
Jensen et al. [33]	- The authors define the variable as “depressive symptoms”. However, during the paper, they also use the term depression. - A cutoff score of 16 to define a clinical, significant level of depression was used.	- The Center for Epidemiologic Studies Depression Scale (CES-D)
Wood et al. [34]	- Depression	- Qualitative research design using interpretative phenomenological - analysis (IPA)
Van Ramele et al. [35]	- Depression	- General Health Questionnaire (GHQ-12)
Sanders and Stevinson [36]	- The authors define the variable as “depressive symptoms”. - Total possible scores range from 0 to 18, with lower scores indicating greater depression.	- Short Depression-Happiness Scale (SDHS; Joseph, Linley, Harwood, Lewis, & McCollam, 2004).
Prinz et al. [37]	- Severity of depression symptoms - Depression	- The Center for Epidemiologic Studies Depression Scale (CES-D) - PHQ-2
Junge and Feddermann-Demont [38]	- The authors define the variable as “depressive symptoms”.	- The Center for Epidemiologic Studies Depression Scale (CES-D)
Gouttebarge et al. [39]	- Depression	- General Health Questionnaire

**Table 5:** A qualitative synthesis of the studies related to burnout.

Study	Aim	Outcomes	Instrument	Main Results
Yildiz, 2015 [40]	Analyse the effect of burnout and bullying in professional players.	Burnout symptoms	Athlete Burnout Questionnaire (ABQ)	Bullying had the strongest and statistically significant direct influence on three dimensions of burnout. (1) reduced sense of accomplishment, (2) emotional/physical exhaustion, and (3) devaluation.
Verardi et al., 2015 [41]	Identify and interpret the occurrence of symptoms associated with burnout syndrome during the pre-competition.	Burnout symptoms	Athlete Burnout Questionnaire (ABQ)	The incidence, and consequently the vulnerability, to burnout, were identified in a portion of the athletes during the pre-competition phase.
Hill, 2013 [42]	Examine the interactive effects of dimensions of perfectionism in predicting symptoms of athlete burnout.	Burnout symptoms	Athlete Burnout Questionnaire (ABQ)	Pure personal perfectionism provided some, albeit limited, protection from burnout in comparison with non-perfectionism. Also, pure evaluative concerns perfectionism, as opposed to mixed perfectionism, emerged as the most debilitating in terms of burnout symptoms.
Curran et al., 2013 [43]	Examine the mediating role of psychological need satisfaction in relationships between types of passion for sport and athlete burnout.	Burnout symptoms and basic psychological need satisfaction	Athlete Burnout Questionnaire (ABQ) and different scales to measure basic psychological need satisfaction	An inverse relationship between harmonious passion and burnout can be explained by higher levels of psychological need satisfaction. However, this was not the case for obsessive passion, which was not associated with psychological need satisfaction or most symptoms of athlete burnout.
Tabei et al., 2012 [44]	Investigate the relationship between organizational stressors in sport and athlete burnout.	Burnout symptoms and organizational stressors	Athlete Burnout Questionnaire (ABQ) and interview	Results revealed multiple demands linked to the dimensions of athlete burnout and identified specific organizational-related issues that players associated with the incidence of burnout.
Yildiz, 2011 [45]	determine whether leader-member exchange quality affects burnout in professional footballers.	Burnout symptoms	An abbreviated version of the 10-item burnout scale	The results demonstrated that the quality of leader-member exchange significantly and inversely influenced burnout of professional footballers
Curran et al., 2011 [46]	To examine the relationship between forms of passion and whether these relationships are mediated by self-determined motivation.	Burnout symptoms, self-determined motivation, and passion	Athlete Burnout Questionnaire (ABQ), Sport Motivation Scale, Passion Scale	The results suggest that harmonious passion may offer some protection from burnout for athletes due to higher levels of self-determined motivation.
